# Repair of bucket handle meniscus tears improves patient outcomes versus partial meniscectomy at the time of ACL reconstruction

**DOI:** 10.1002/jeo2.70004

**Published:** 2024-08-28

**Authors:** Gregory T. Perraut, Rachel E. Cherelstein, Alexandra M. Galel, Laura E. Keeling, Christopher M. Kuenze, Andrew J. Curley, David X. Wang, Kaitlin A. Malekzadeh, Edward S. Chang

**Affiliations:** ^1^ Department of Orthopedics Georgetown University Medical Center Washington District of Columbia USA; ^2^ Inova Sports Medicine Fairfax Virginia USA

**Keywords:** ACL reconstruction, bucket‐handle meniscus tear, knee arthroscopy, meniscal repair, meniscectomy

## Abstract

**Purpose:**

The aim of this study was to examine demographic and surgical factors that influence patient‐reported knee function in patients who undergo anterior crucial ligament reconstruction (ACLR) with concurrent bucket‐handle meniscal tear (BHMT) procedures. We hypothesized that repair of BHMT in the setting of concomitant ACLR and shorter time from injury to surgery would lead to improved patient‐reported outcomes.

**Methods:**

Forty‐one patients (mean age: 28.0 ± 9.8 years, 72% male) with BHMT at the time of ACLR completed the International Knee Documentation Committee Subjective Knee Form (IKDC‐SKF) via online survey at an average of 15.2 months postop. Patient demographics and surgical characteristics, including time from injury to surgery, were compared between repair (*n* = 22) and meniscectomy (*n* = 19) groups using one‐way analysis of variances; distributions of sex, graft source, BHMT compartment and zone were compared between groups using *χ* ^2^ tests. The association between IKDC‐SKF score, demographics and surgical characteristics was evaluated using multivariable linear regression. A priori alpha level was *p* < 0.05.

**Results:**

Meniscal repair and meniscectomy groups differed based on graft source and BHMT zone but not IKDC‐SKF score (*p* = 0.085). Patients undergoing ACLR with autograft (*p* = 0.003) and with red–red zone BHMT (*p* < 0.001) more often underwent meniscal repair. The regression model demonstrated longer time from injury to surgery (*p* = 0.049), red–red tear zone (*p* = 0.04) and meniscectomy (*p* = 0.008); these were predictive of poorer IKDC‐SKF scores.

**Conclusion:**

BHMT repair was more likely performed in ACL autograft and on red–red zone tears. Longer time from injury to surgery is an indicator of poorer IKDC‐SKF score, as this may increase the risk of concomitant pathologies. White–white zone BHMTs are associated with better IKDC‐SKF scores than red–red zone BHMTs, which may be due to the smaller volume of tissue removed during meniscectomy of white–white zone tears and the avoidance of iatrogenic complications of meniscal repair.

**Level of Evidence:**

Level III, therapeutic study.

**Abbreviations**
ACLR: ACL reconstruction
BHMT: bucket‐handle meniscus tear
BMI: body mass index
IKDC‐SKF: International Knee Documentation Committee Subjective Knee Form
KOOS: Knee Injury and Osteoarthritis Outcome Scores

## INTRODUCTION

The meniscus serves an important function in load distribution, shock absorption and stability of the knee joint. Injuries to the meniscus frequently occur with tears of the anterior crucial ligament (ACL), ranging from 51.9% to 63% [14]. Treatment of meniscal injuries in the setting of ACL tears is further complicated by the presence of a bucket‐handle meniscus tear (BHMT) [11, 23]. These injuries present a unique challenge to the surgeon due to their complexity attributed to their increased size, significant displacement and variability in blood supply based on location of the tear [17].

Current practice now emphasizes preservation of the meniscus, especially in the setting of a concomitant ACL tear. Multiple studies have demonstrated the chondroprotective effect and improved stability with meniscus preservation and concurrent ACL reconstruction (ACLR) [5, 13, 15, 17]. Kalifis et al. found that patients who had successful repair of BHMT led to lower rates of osteoarthritis and better knee function up to 10 years postoperatively compared to patients with a failed BHMT repair [13].

The decision to perform meniscectomy or repair of BHMT relies on many factors, including size of tear, vascular location and chronicity of tear. The meniscus is known for its peripheral blood supply, which makes healing of some tears very difficult [2]. The outer third, well‐vascularized region of the meniscus is known as the red–red zone, while the poorly vascularized inner third is termed the white–white zone. The middle third of the meniscus creates the red–white zone. Traditionally, BHMTs in the red–red region are repaired while those in the white–white zone are debrided, as red–red zone tears have a much higher likelihood of healing compared to the other zones [8, 9]. However, many other demographic factors such as age, smoking status, body mass index (BMI), concurrent ACL tear and time from injury to surgery are also considered [25].

While there have been multiple studies examining outcomes following isolated BHMT, less is known about factors influencing outcomes following ACLR and concurrent BHMT. To our knowledge, this is the first paper to analyse patient outcomes after ACLR with concomitant BHMT. The purpose of this study was to examine demographic and surgical factors that influence patient‐reported knee function in patients who undergo ACLR with concurrent BHMT procedure, either meniscectomy or repair. We hypothesized that repair of BHMT in the setting of concomitant ACLR and shorter time from injury to surgery would lead to improved patient‐reported outcomes.

## METHODS

This was a retrospective cohort study investigating postoperative outcomes for BHMT in patients undergoing concomitant ACLR. Patients were identified via a retrospective chart review and were subsequently contacted by the research team for collection of outcome data. This study was approved by the WCG Institutional Review Board, and patients provided written informed consent.

### Participants

**Figure 1:**
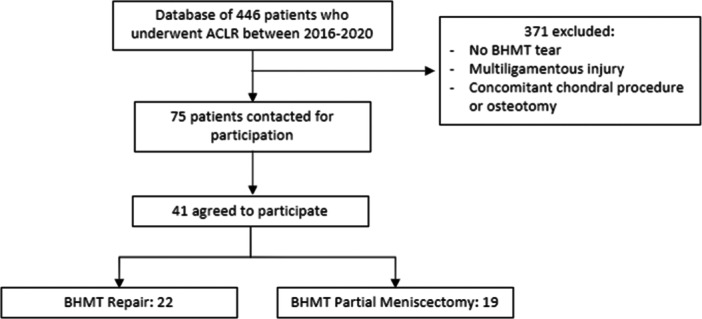
CONSORT flow diagram illustrating grouping of patients.

All patients who underwent ACLR with concomitant surgical treatment of a BHMT from December 2015 through February 2020 were contacted. Exclusion criteria included patients presenting with multi‐ligamentous knee injury, defined as surgical repair or reconstruction of more than one ligament, and those undergoing concomitant chondral procedures or osteotomy. All surgeries were performed by one of five sports medicine fellowship‐trained orthopaedic surgeons. Patients were divided into two groups, including those who underwent partial meniscectomy and those who had meniscus repair at the time of ACLR. Patients with less than 1 year of follow‐up were subsequently excluded from the final analysis. From a database of 446 patients who underwent ACLR between 2016 and 2020, 75 patients were identified as having surgery to address a concomitant BHMT. Forty‐one patients agreed to participate in an average follow‐up of 15.2 months. Of those, 19 patients underwent partial meniscectomy while 22 underwent meniscal repair (Figure 1).

### Surgical Technique

#### ACLR

All patients underwent ACLR with a standardized procedure. For QT autograft harvest, a 4 cm incision was made superior to the superior patellar pole, and sharp dissection was carried down to the paratenon to harvest 7 cm of all‐soft tissue tendon. For BTB autograft harvest, an incision was made over the medial border of the patellar tendon, and the patellar tendon was harvested at a width of 10 mm. A sagittal saw was used to create 20 mm × 10 mm bone plugs from both the patella and the tibia. For HT autograft harvest, a 4 cm incision was made between the tibial tubercle and the medial tibia, and a 9 mm quadrupled semitendinosus graft was harvested. In all cases, autografts were soaked in an antibiotic‐soaked sponge and prepared with either suspensory or aperture fixation, based on surgeon preference.

Following diagnostic arthroscopy and any necessary meniscal procedures, the ACL stump was gently debrided, leaving a small remnant on both the tibial and femoral footprint. Independent femoral tunnels were created in an anterograde with the knee hyperflexed or retrograde manner. For the tibial tunnel, a drill guide was placed in the centre of the ACL footprint, and the tunnel was created in an anterograde fashion. Both tibial and femoral tunnels were reamed using appropriately sized reamers based on graft width. Following graft passage, femoral fixation was achieved either with an interference screw or a cortical button, based on surgeon preference. The knee was then cycled to remove excess creep, and a tibial fixation was secured with the knee in extension and a posterior drawer applied. All patients were then placed in a hinged knee brace locked in extension before being woken from anaesthesia.

#### Partial meniscectomy

For BHMTs that were not deemed reparable, a standard partial meniscectomy was performed using a series of arthroscopic biters and shavers. Irreparable tears were classified as chronic tears, those that were irreducible, complex tear patterns and those confined predominantly to the white–white zone. A minimal amount of tissue possible was removed to preserve the maximum amount of meniscal tissue.

#### Meniscus repair

BHMTs that were deemed reparable were repaired in an all‐inside manner using an all‐inside meniscal repair device (FAST‐FIX 360, Smith & Nephew) in a mattress fashion. These were tears involving the red–red zone and red–white zone predominantly and were adequately reducible. The number of devices used varied based on tear size.

### Chart review

Demographic information retrieved via retrospective chart review included BMI, age, smoking status and time from injury to surgery. If a patient was unable to recall the exact date of their injury, a date was calculated based on their best estimation of the time frame stated on the date of their initial visit. Clinical information collected via review of operative notes included BHMT compartment and zone.

### Patient‐reported knee function

Participants completed the International Knee Documentation Committee Subjective Knee Evaluation form (IKDC‐SKF) electronically. The IKDC‐SKF is used to measure symptoms, function and sports activity for people with a variety of knee disorders, including ligamentous and meniscal injuries, osteoarthritis and patellofemoral dysfunction [12]. It has been shown to allow physicians to interpret how patients with knee injuries above the age of 18 years are functioning relative to their age‐ and gender‐matched peers [1].

### Statistical analysis

Descriptive statistics were calculated for patient demographics, injury and surgical characteristics and IKDC‐SKF score. Continuous variables were compared between the repair and meniscectomy groups using one‐way analysis of variances while the distributions of biologic sex, graft source, bucket handle tear compartment and zone were compared between groups using *χ* ^2^ tests. Multivariable linear regression was used to determine the association between patient‐reported knee‐related function and patient demographics, injury characteristics and meniscus treatment characteristics. A priori alpha level was established as *p* < 0.05, and all statistical analyses were completed in Jamovi v 2.2.5. open‐source statistical software.

### Post hoc power analysis

A post hoc power analysis was performed given the modest sample size in this study. Given the *R* ^2^ value (0.366), five predictor variables and an acceptable power (1 − *β*) of 0.80, a minimum of 29 participants was needed to develop this model with a low risk of spurious statistical findings.

## RESULTS

### Between‐group comparison

Patient baseline characteristics, including comparisons between groups, are summarized and presented in Table 1. A total of 41 patients (28.0 ± 9.8 years; range, 14–53 years) were included in the analysis, with 72% of patients being male. There were 22 patients in the repair cohort and 19 patients in the meniscectomy cohort. The mean follow‐up time was 15.2 months. The meniscal repair and meniscectomy groups differed based on graft source (*p* = 0.003) and meniscus tear zone (*p* < 0.001), with 13 of 15 red–red tears undergoing repair and 10 of 12 white–white zone tears undergoing meniscectomy.

**Table 1:** Comparison of demographics and injury characteristics between patients treated with meniscus repair and those treated with meniscectomy.

	Meniscus repair	Meniscectomy	*p* Value	Cohen's *d* (95% CI)
Biologic sex (male, female)	14, 8	13, 6	0.75	—
Age (years)	29.8 ± 10.0	27.2 ± 10.2	0.403	0.268 (−0.362, 0.891)
Body mass index (kg m^−2^)	27.1 ± 5.9	29.4 ± 5.7	0.208	0.405 (−0.234, 1.03)
Time from injury to surgery (years)	2.22 ± 5.32	1.60 ± 2.72	0.499	0.216 (−0.411, 0.838)
Time from ACLR to follow‐up (years)	1.28 ± 0.95	1.21 ± 0.87	0.672	−0.135 (−0.756, 0.489)
Graft source	11 BPTB Auto 7 HS Auto 0 HS Allo 0 Posterior Tib Allo 2 QT Auto 2 Tibialis Ant Auto	2 BPTB Auto 11 HS Auto 4 HS Allo 1 Posterior Tib Allo 0 QT Auto 1 Tibialis Ant Auto	0.003	—
Bucket handle tear compartment	4 Lateral 17 Medial	2 Lateral 17 Medial	0.67	—
Bucket handle tear zone	13 Red–Red 6 Red–White 2 White–White	2 Red–Red 4 Red–White 10 White–White	<0.001	—
IKDC‐SKF Score (0–100)	78.7 ± 21.2	71.9 ± 18.5	0.085	−0.560 (−0.1.19, 0.094)

Abbreviations: ACLR, ACL reconstruction; Allo, allograft; Auto, autograft; BPTB, bone‐patellar tendon‐bone; HS, hamstring; IKDC‐SKF, International Knee Documentation Committee Subjective Knee Form; Posterior tib, posterior tibialis tendon; QT, quadriceps tendon; Tibialis ant, tibialis anterior tendon.

The mean IKDC‐SKF score at the final follow‐up was 71.9 ± 18.5 in the meniscectomy cohort and 78.7 ± 21.2 in the repair cohort. No significant differences were noted in IKDC (*p* = 0.085) scores between the repair and meniscectomy cohorts at follow‐up when directly compared with no other variables taken into consideration. Of note, repair and meniscectomy cohorts did not differ in time from ACLR to IKDC‐SKF follow‐up (*p* = 0.672).

One patient in the meniscectomy group (5.00%) experienced a retear of the ACL 2 years following the index surgery, while at the most recent follow‐up no patients in the repair group had experienced an ACL retear. One patient in the meniscal repair group (7.14%) experienced a recurrent BHMT, while no patients in the meniscectomy group experienced a recurrent meniscal tear at the final follow‐up.

### Regression analysis

A regression model that included graft source, time from injury to ACLR, time from ACLR to IKDC‐SKF, tear zone and tear treatment predicted 36.6% of variance in IKDC‐SKF score among this cohort (Table 2). In this regression analysis, lower time from injury to ACLR (*p* = 0.028), white–white tear zone (*p* = 0.040) and meniscal repair (*p* = 0.008) were significant predictors of greater IKDC‐SKF scores after accounting for the other variables in the model.

**Table 2:** Demographic, injury and surgical predictors of patient‐reported knee‐related function.

Predictor	Estimate	SE	*t*	*p*
Intercept	59.64	6.06	9.84	<0.001
Graft source				
Allograft–Autograft	6.42	6.45	1.00	0.328
Time from injury to ACLR (days)	−0.004	0.01	−2.32	0.028
Time from ACLR to IKDC‐SKF (days)	0.24	0.01	3.15	0.004
Bucket handle tear zone				
Red:White–Red:Red	3.29	6.24	.53	0.602
White:White–Red:Red	16.61	7.71	2.16	0.040
Bucket handle tear treatment				
Repair—Meniscectomy	18.87	6.66	2.83	0.008

While these findings may initially appear to contradict the between‐group comparisons between the repair and meniscectomy cohorts (Table 1), it should be noted that the regression analysis takes into consideration all included variables, as indicated in Table 2. Therefore, while meniscal repair and meniscectomy cohorts did not differ in IKDC‐SKF score when directly compared, meniscal repairs predicted better IKDC‐SKF scores when considering variation in other variables.

## DISCUSSION

The results of this study indicate that among patients presenting with concomitant bucket‐handle meniscus and ACL tears, better IKDC‐SKF scores are predicted by meniscal repair and shorter time from injury to ACLR. The results also found that BHMTs in the white–white zone demonstrated improved IKDC‐SKF scores compared to red–red zone tears. This demonstrates that whenever possible, the meniscus should be preserved and repaired. However, in cases of chronic, complex and predominantly white–white zone tears, these are indicated for meniscectomy due to the decreased healing capability of these tears. Performing a meniscectomy of a small volume of tissue rather than repair on these select cases will allow them to weight‐bear faster and decrease the risk of retearing or repair failure and a potential second surgery.

While meniscectomy is still the most common procedure performed for meniscal tears [18, 21], meniscal preservation, when possible, is preferred especially in the setting of ACLR [6, 19, 22]. The potential benefit in meniscal repair includes possible delay in osteoarthritic changes in knees with preserved meniscal function, as well as evidence of unsatisfactory outcomes in terms of pain, stiffness and range of motion in patients who had undergone meniscectomy [19]. The results indicate that meniscus repair does appear to be associated with improved knee function in patients undergoing ACLR with a concomitant BHMT. These findings also suggest that other factors may play a role in improved patient knee function, including shorter time from injury to surgery and zone of tearing within the meniscus. These results can help surgeons guide their treatment of patients presenting with BHMT in the setting of ACL tears, as multiple factors should be taken into consideration with each patient to provide them with the best possible functional outcome.

It has been widely accepted that meniscus preservation is key in preventing or delaying long‐term sequelae of impaired tibiofemoral biomechanics and the development of osteoarthritis [13, 15, 17, 22]. Kalifis et al. reported that BHMT repair can be associated with a high rate of failure, noting a failure rate of 33% in a study of 66 knees with BHMTs. Successful repairs were associated with significantly higher Knee Injury and Osteoarthritis Outcome Scores (KOOS), IKDC‐SKF score and Lysholm score compared to failed repairs. In addition, successful repairs had a significantly lower rate of osteoarthritis. While this study did not examine rates of degeneration or osteoarthritis, the data do support previous literature demonstrating superior patient outcome scores after meniscal repair.

This study also suggests that worse outcomes after BHMT repair in the setting of concomitant ACLR were associated with longer time between injury and surgery. This may be due to the higher likelihood of concomitant pathologies seen with increased time between injury and surgery in ACLR with associated meniscus tears. Prior literature has documented increased risk of high‐grade cartilage lesions, medial meniscus tears, new meniscal tears and overall degenerative changes the longer surgery is delayed [3, 16]. However, it should be noted that multiple studies have shown acceptable outcomes following delayed repair of BHMTs. Espejo‐Reina et al. found repair of chronic BHMT leads to good postoperative outcomes with a failure rate of 17% [7]. Moatshe et al. studied differences in outcomes between repair techniques of BHMT versus vertical meniscus tears as well as difference between acute and chronic tears. Interestingly, they found no differences in outcomes in repairs of acute versus chronic BHMT. The authors concluded that repair of BHMT can be attempted even in chronic cases. Further studies concerning timing of BHMT repair in patients with concomitant ACL tear should be performed to determine the proper treatment plan for these patients.

There was an increased tendency towards repair of tears in the red–red zone (13/15 repaired) compared to those in the white–white zone (2/12 repaired). While this may not be surprising given the healing potential can depend on vascularity zones [4, 20, 24], what we found interesting was that patients with white–white zone tears were associated with improved outcomes as compared to red–red zone tears. As mentioned earlier, the treatment between the two groups was different, with most red–red zone tears undergoing repair and white–white zone tears undergoing meniscectomy. And while the regression analysis demonstrates that repairs in general have a higher IKDC‐SKF score, a white–white zone tear treated with a meniscectomy can have a comparable, if not better, IKDC‐SKF score. This can potentially be explained by the fact that white–white tears are generally smaller and, therefore, less volume of tissue is removed during meniscectomy. The large volume of intact tissue can then continue to provide stability and protection to the knee. Another possible explanation is that white–white zone tears treated with meniscectomy do not have to deal with the potential adverse side effects of meniscal repair (iatrogenic injury, implant‐related complications, re‐tearing of the meniscus, delayed weight‐bearing) [10].

This study has several strengths. First, the results are generalizable as the cohort reflects the average patient that sustains BHMT: young males with medial meniscus involvement more commonly than lateral. Second, all BHMT repairs were performed with an all‐inside technique, which minimizes another potential variable. Third, we have a follow‐up period of a minimum of 15 months to capture potential perioperative complications and assess patient satisfaction.

The study is not without limitations. First, this was a retrospective study and therefore is prone to bias, particularly recall bias, and confounding factors commonly seen in this study design. Given the retrospective nature of the study and results collected via online survey, we were unable to collect objective clinical outcomes and chose to focus on patient‐reported outcomes via IKDC scores. Prospectively collected objective clinical outcomes would be an important direction for future research on this topic. The population was significantly different based on graft source. This variability is based on surgeon and patient preference during preoperative discussion, and we believe it does help with the generalizability of the study. However, the regression analysis did not find graft source to be a significant predictor of patient outcomes; thus, we do not believe this to be a confounding factor. Additionally, we were not able to consider the size of meniscal tears or outerbridge scores of patients due to the retrospective nature of the study. The study also included patients operated on by five different surgeons, which may lead to confounders regarding graft choice and definition of repairable versus irreparable meniscal tears, though we believe this enhances the generalizability of the study to the population. The sample size is relatively small, with a total of 41 patients participating in the study, which may lend us to an underpowered study, though the post hoc power analysis does not support this. Nonetheless, a prospective study will help to support these findings. Lastly, the study reported on early to mid‐term follow‐up with an average follow‐up of 15.2 months, and therefore, it is possible that we may have missed some retears.

## CONCLUSION

This study showed that patients with BHMTs in the setting of ACL tears are predicted to have better postoperative outcomes with meniscal repair rather than meniscectomy. In addition, better outcomes are predicted by shorter time to surgery from injury and tear location within the white–white zone, which may be more amenable to meniscectomy. It is critical to consider tear location and complexity, as those with poor healing potential will likely perform better with meniscectomy rather than repair. Overall, surgeons should attempt repair of BHMTs in the setting of concomitant ACL tears whenever feasible to improve patient's postoperative outcomes and to promote knee preservation and reduce the risk of osteoarthritic changes within the knee.

## AUTHOR CONTRIBUTIONS

All authors contributed to the study conception and design. Material preparation, data collection and analysis were performed by Gregory T. Perraut, Rachel E. Cherelstein, Alexandra M. Galel, Laura E. Keeling, Christopher M. Kuenze, Andrew J. Curley, David X. Wang, Kaitlin A. Malekzadeh and Edward S. Chang. The first draft of the manuscript was written by Kaitlin A. Malekzadeh and Gregory T. Perraut, and all authors commented on previous versions of the manuscript. All authors read and approved the final manuscript.

## CONFLICT OF INTEREST STATEMENT

The authors declare no conflict of interest.

## ETHICS STATEMENT

Ethical approval for this study was obtained from the WCG Institutional Review Board. Written, informed consent was obtained from all patients included in the study.

## Data Availability

The data that support the findings of this study are available from the corresponding author upon reasonable request.
